# Fatty Acids and Membrane Lipidomics in Oncology: A Cross-Road of Nutritional, Signaling and Metabolic Pathways

**DOI:** 10.3390/metabo10090345

**Published:** 2020-08-25

**Authors:** Carla Ferreri, Anna Sansone, Rosaria Ferreri, Javier Amézaga, Itziar Tueros

**Affiliations:** 1 Istituto per la Sintesi Organica e la Fotoreattività, Consiglio Nazionale delle Ricerche, Via Piero Gobetti 101, 40129 Bologna, Italy; anna.sansone@isof.cnr.it; 2 Department of Integrated Medicine, Tuscany Reference Centre for Integrated Medicine in the hospital pathway, Pitigliano Hospital, Via Nicola Ciacci, 340, 58017 Pitigliano, Italy; rosariaferreri1957@gmail.com; 3 AZTI, Food and Health, Parque Tecnológico de Bizkaia, Astondo Bidea, Edificio 609, 48160 Derio, Spain; jamezaga@azti.es (J.A.); itueros@azti.es (I.T.)

**Keywords:** cancer cell membranes, fatty acid biosynthesis, essential fatty acids, desaturase enzymes, fatty acid signaling, fatty acid biomarker, sapienic acid, sebaleic acid, molecular nutrition, inflammation

## Abstract

Fatty acids are closely involved in lipid synthesis and metabolism in cancer. Their amount and composition are dependent on dietary supply and tumor microenviroment. Research in this subject highlighted the crucial event of membrane formation, which is regulated by the fatty acids’ molecular properties. The growing understanding of the pathways that create the fatty acid pool needed for cell replication is the result of lipidomics studies, also envisaging novel fatty acid biosynthesis and fatty acid-mediated signaling. Fatty acid-driven mechanisms and biological effects in cancer onset, growth and metastasis have been elucidated, recognizing the importance of polyunsaturated molecules and the balance between omega-6 and omega-3 families. Saturated and monounsaturated fatty acids are biomarkers in several types of cancer, and their characterization in cell membranes and exosomes is under development for diagnostic purposes. Desaturase enzymatic activity with unprecedented *de novo* polyunsaturated fatty acid (PUFA) synthesis is considered the recent breakthrough in this scenario. Together with the link between obesity and cancer, fatty acids open interesting perspectives for biomarker discovery and nutritional strategies to control cancer, also in combination with therapies. All these subjects are described using an integrated approach taking into account biochemical, biological and analytical aspects, delineating innovations in cancer prevention, diagnostics and treatments.

## 1. Introduction

**Figure 1:**
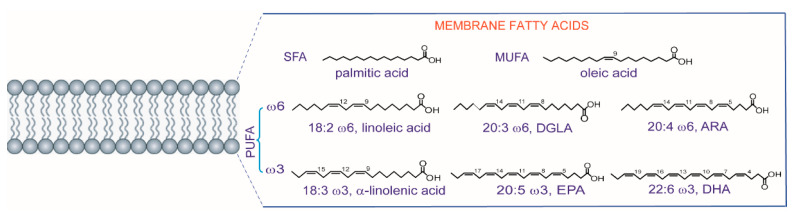
The fatty acid constituents of phospholipids: saturated fatty acids (SFA), monounsaturated fatty acids (MUFA) and polyunsaturated fatty acids (PUFA) are shown with their most present structures in eukaryotic membranes.

**Figure 2:**
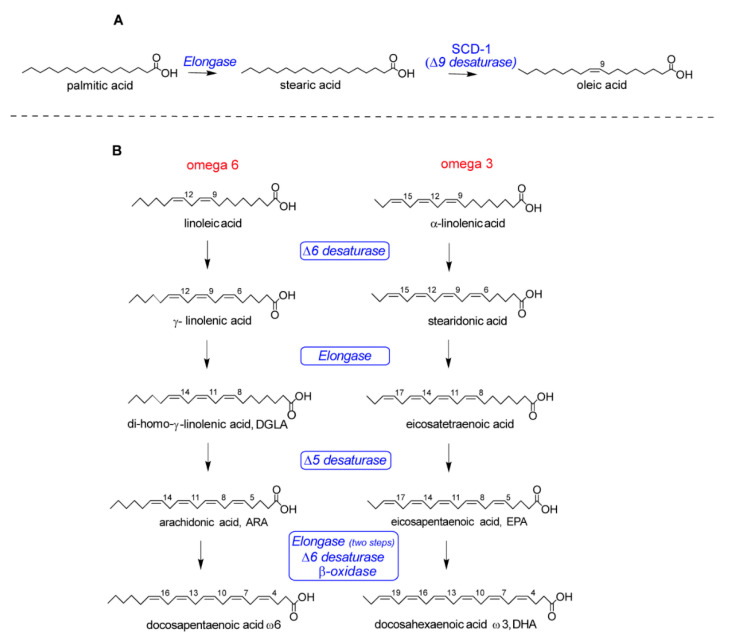
Some metabolic transformations of fatty acids: (**A**) the saturated fatty acid (SFA), palmitic acid, is transformed into stearic acid and the monounsaturated fatty acid (MUFA), oleic acid; (**B**) omega-6 and omega-3 precursors taken from the diet are transformed into the other polyunsaturated fatty acids (PUFA) members of the two families.

The development of lipid research in the last two decades has brought a fundamental contribution to the understanding of the main processes for cellular life, in all types of organisms as well as in plants [1]. In particular, fatty acids are the building blocks of the large majority of lipid structures, differentiated from lipids that have steroid and isoprenoid scaffolds. Fatty acids are known for their multiple roles, ranging from energy providers and gene regulators to precursors of signaling molecules and other important metabolites, but it is worth noting that fatty acids in phospholipids have specific structural and functional roles in order to create the envelope of all types of cells, i.e., the cell membrane [2]. In eukaryotes, fatty acids display structural diversity and, as represented in Figure 1 with the most important molecules for the organization of membrane phospholipids, are characterized by specific chain length and number of unsaturations. First of all, the length of the hydrocarbon (hydrophobic) chains requires a certain number of carbon atoms (most often 16–22 carbon atoms) to create the membrane compartment and the thickness of the lipid bilayers. Biosynthesis is initiated with the formation of 16 carbon atoms containing palmitic acid, the first endogenous lipid which is a saturated fatty acid (SFA) (Figure 1) made by the enzymatic system of fatty acid synthase (FAS). Together with the chain length, another structural requirement present in unsaturated fatty acids is the geometry of cis double bonds. The enzymatic system of desaturases introduces the unsaturation in a precise position of the fatty chain (indicated with the carbon atom number; see Figure 1) and this creates a bend (angle of ca. 30 degrees), modifying profoundly the biophysical properties of the molecules [3]. The main endogenous formation of double bonds is due to delta-9 (Δ9) desaturase (also known as stearoyl CoA desaturase SCD-1) operating on palmitic and stearic acids, as shown in Figure 2A.

On the other hand, the polyunsaturated fatty acid (PUFA) structures are necessary to eukaryotic cells but are not biosynthesized *de novo*, and the precursors of the omega-6 and omega-3 families must be taken from the diet. The structures of the omega-6 and omega-3 precursors are shown in Figure 1 (linoleic acid and α-linolenic acid, respectively) and, after their uptake, other PUFAs are formed and enter into the membrane composition, as shown in Figure 1. In Figure 2B, the two pathways followed for long-chain PUFA biosynthesis are shown, with formation of omega-6 di-homo-gamma linolenic (DGLA) and arachidonic (ARA) acids and omega-3 eicosapentaenoic (EPA) and docosahexaenoic (DHA) acids. As a matter of fact, the transformation to mono- and polyunsaturated fatty acids (MUFA and PUFA) provides the precious building blocks of membrane phospholipids involved in the regulation of permeability and fluidity properties. MUFAs and PUFAs act in a manner opposite to SFA, which instead create the rigidity and the gel status of the lipid bilayer. The role of fats in cancer is generally recognized [4], and the SFA-MUFA pathway has been studied since it is one of the pieces of the puzzling scenario for tumoral cell development and invasion [5]. However, considering that the membrane is necessary for cell formation and reproduction, the ways in which the balance among SFAs, MUFAs and PUFAs influences these steps are still to be defined. MUFAs can be obtained totally by an endogenous process, whereas PUFAs cannot be biosynthesized in humans, as shown in Figure 2B. Due to this “dietary dependency”, the effects of an impairment of both exogenous supply and endogenous metabolism needs a comprehensive approach in order to examine cellular metabolism, signaling and nutrition. This is why fatty acid-based membrane lipidomics drives important information in health and diseases and, in particular in cancer “-omics”, it is needed for the comprehension of molecular mechanisms and for biomarker discovery.

Here we wish to remark that a multidisciplinary approach is necessary, where chemical, biological and clinical skills are required all at once. Indeed, in membrane lipids research and medical applications, all these skills are also necessary to address critical issues in protocols: are we fully conscious of the difference in monitoring circulating lipids from those entering the cell membrane composition? Can we make crucial decisions about what is the best sampling procedure for cell membrane lipids? Finally, can we make an effort to unify protocols in one accredited procedure, so that big data can be collected and results can be compared in multicentric studies? In our opinion, analytical and chemical competences here come first, since they are required in order to build up accurate and reliable protocols: the recognition of fatty acid structures must be unambiguous [6], as will be shown in this review, taking into account that fatty acids are tissue-specific and each tissue has its own distribution of these molecular components [7]. Quality control must involve the exact separation of fatty acids from the sample to be analyzed, and if membranes are the target, the procedure must isolate them. This accuracy is fundamental because, after analysis, fatty acids are interpreted for their biological effects, as precursors to lipid mediators and contributors to membrane fluidity.

The contribution of fatty acids to membrane properties has been recognized for a long time, particularly in cancer development [8]. More recently, it has been discussed as evidence that the application of membrane modification and manipulation as part of cancer therapeutical strategies is still not developed [9].

An interplay between biosynthesis and diet regulates fatty acid availability. We gathered the literature on how fatty acids are implicated in tumor onset and progression and how the cancer lipidome reflects the activation of the *de novo* synthetic pathways. In this overview, we wish also to highlight our own work on the discovery of a family of MUFA positional isomers, the n-10 family, as new biomarkers of the metabolic shift that allows human cells to build up the first endogenous PUFA component, sebaleic acid [10]. The review also covers the link between obesity and cancer in order to understand why and when lipid supply causes health complications, highlighting specific fatty acids for their biological effects, signaling and contribution to the membrane properties that influence cell growth and death. From this scenario, several hints emerge for innovative strategies in cancer prevention (primary and secondary) using fatty acid-based membrane lipidomics and fatty acid balance.

## 2. Fatty Acids and Lipid Supply for Membrane Formation in Cancer

Cancer is a very complex disease due to the large number of factors involved. Cells develop a great capacity to grow, proliferate and survive under stress conditions. They modify several processes to achieve favorable environments, such as the metabolism of lipids, carbohydrates, proteins and nucleotides, being able to maintain the functionality of the structures and functions [11,12]. Adapted metabolic pathways allow cancer cells to obtain energy, form metabolic intermediates and synthesize fatty acids, even when the exogenous availability of these compounds is reduced. For example, the hyperactivation of the phosphatidylinositol-3 kinase and AKT (PI3K-AKT) transduces the signal from the hormone insulin to drive glucose uptake and is one of the most frequently mutated pathways in cancer [13]. In this case, glycolysis is favored, leading the cells to form pyruvate, which could be used for ATP synthesis or for *de novo* lipogenesis [14]. The hyperactivation of PI3K-AKT also activates the glutamate pyruvate transaminase 2 (GPT2), favoring glutamine anaplerosis to supply sufficient metabolites for FA synthesis and, finally, remodel the cellular lipidome [15]. In the latter case, it has been shown that such remodeling makes lipids an important hallmark of cancer [16]. The overexpression of FA transporters, such as fatty acid translocase CD36, plasma membrane fatty acid-binding proteins (FABP) and the fatty acid transport protein family (FATP), elevates the uptake of exogenous FAs with their subsequent storage in lipid droplets (LDs), as is known in ovarian cancer, and this is in connection also with adipose tissue, as will be explained in Section 5 [17]. To fully evaluate the lipid supply and understand their role in cancer, we must distinguish between *de novo* synthesized and dietary fatty acids, as explained below.

### 2.1. De Novo Synthesis of Saturated and Monounsaturated Fatty Acids

**Figure 3:**
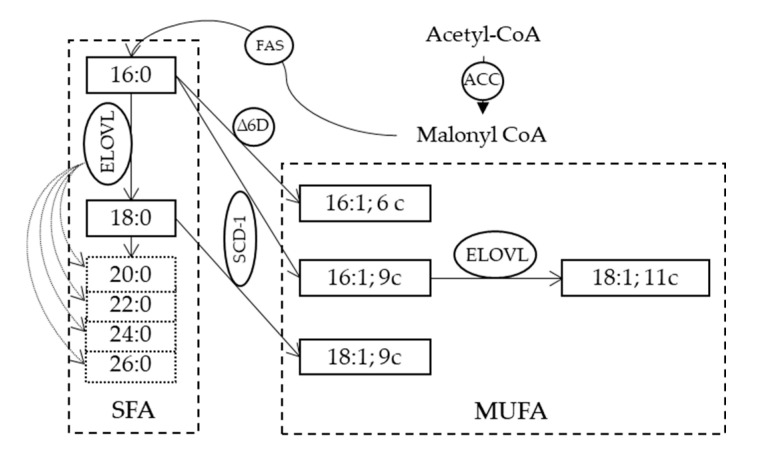
The *de novo* synthesis of saturated fatty acids (SFA) starting from acetyl CoA and the transformation to monounsaturated fatty acids (MUFA) by two desaturase enzymes. Structures of some of these fatty acids are shown in Figure 1. ACC: acetyl CoA: carboxylase; FAS: fatty acid synthase; ELOVL: elongase enzyme; Δ6D: delta-6 desaturase (Δ6); SCD-1: stearoyl CoA desaturase.

Combined with a greater capacity for the biosynthesis of lipids, cancer cells are not only able to maintain lipid homeostasis but also to provide ATP and NADPH in conditions of metabolic stress and sufficient precursors to deal with the formation of lipid rafts that are essential for protein dynamics in membranes and cell survival [18,19]. Since phospholipids are the basic units of membranes, in cancer disease different enzymes involved in their endogenous synthesis are highly expressed, such as ATP-citrate lyase (ACLY), acetyl CoA carboxylase (ACC) and fatty acid synthase (FAS) [20,21]. Each of them represents itself a target of study against cancer. Whereas in nutrient-unlimited and aerobic conditions, the glucose metabolism forms citrate through the tricarboxylic acid cycle (TCA), to later convert into acetyl CoA, being the key for *de novo* synthesis, cells also develop an alternative strategy to form FA when there is a lack of nutrients and hypoxia (Figure 3). Several groups have proposed that, in these mentioned cases, the TCA cycle can be modified to run in reverse and use glutamine from storage to act as source of acetyl CoA [22,23], whereas others describe that acetyl CoA can be obtained from histone deacetylation [24]. In the case of FAS, in addition, it responds to signals from the activation of the AKT and MAPK (mitogen-activated protein kinase) pathways, which, in turn, are also favored in cancer processes [25]. Besides the fact that the production of FAs is essential to sustain the structure and demands of membranes, their composition is also decisive in guaranteeing the functions of the dividing cells. The production of monounsaturated fatty acids (MUFA) from SFA provides fluidity, functionality and flexibility, which are essential for tumor cells. This step involves the action of delta-9 desaturase enzyme (known also as stearoyl CoA desaturase, SCD-1, and reported in Figure 3), which can act on both palmitic (16:0) and stearic (18:0) acids. As with the enzymatic complex from *de novo* synthesis, SCD-1 is overexpressed in cancer and regulated by different signaling cascades such as MAPK and AKT or systems such as p53 [26], attracting interest in their inhibition [4,5].

Cancer cells modify lipid metabolism in order to respond to environmental modifications. Hypoxia, for example, affects acetyl CoA formation from glucose and SCD-1 activity, as they are oxygen dependent. However, in this case, tumoral cells escape the need for fatty acid synthesis by increasing the uptake of lysophospholipids as a shortcut to prepare phospholipids. FABPs are transcriptional targets of hypoxia-inducible factors (HIFs) that facilitate extracellular scavenging of long-chain unsaturated lysophospholipids, which can be used as a nutrient source under conditions of metabolic stress [15]. Interestingly, this effect can occur even in aerobic conditions after oncogenic RAS activation, making it independent from SCD-1, to achieve sufficient MUFAs [27]. It is worth highlighting the existing debate about whether fatty acids used by cancer cells are of endogenous or exogenous (dietary) origin, since some studies did not find differences [28]. Lipidomic studies have a fundamental role in the elucidation of the decisive contribution of fatty acid biosynthesis, evidencing storage, lipolysis and membrane remodeling implied in tumor onset, progression and metastasis. Table 1 summarizes the most important fatty acid-driven mechanisms and related biological effects.

Readers are directed to the original references cited in Table 1 to elaborate on each subject appropriately. Among these mechanisms, a recent one (Entry 1) was individuated by some of our group, investigating the analytical protocols for efficient separation of the MUFA positional isomers, which will be addressed in Section 4. Knowledge of different mechanisms is necessary for research of new therapeutic targets that can act in a synergic manner, to disturb organization of membrane lipids, destabilize lipid rafts and activate apoptosis signaling [18,19].

### 2.2. PUFA Intake and Omega-6/Omega-3 Balance for Membrane Fatty Acid-Mediated Signaling

On the basis of the importance of phospholipids for cell formation, the “membrane hypothesis” can be drawn, for which the initial steps of death or life of tumoral cells could be also driven by the quality of the membrane fatty acids. To create a fatty acid balance among SFA, MUFA and PUFA residues in the individual, it must be taken into account that the dietary intake of omega-6 and omega-3 regulates the presence of PUFA residues in lipid pools. Once the individual pool is formed, it exerts strong control upon the membrane composition and the types of fatty acids that will be detached from membrane phospholipids to determine the related cell fate. Indeed, the phospholipase A_2_ (PLA_2_)-induced release of fatty acids from membranes is a well-known process, involved in the membrane remodeling cycle, i.e., the Lands cycle [40]. It does not discriminate between omega-3 and omega-6 structures, thus highlighting the importance of the above-mentioned balance present in membranes for pro- and anti-inflammation signaling. Indeed, every time that the release in the cytoplasm of arachidonic acid from phospholipids occurs by PLA_2_, causing the subsequent formation of its eicosanoid mediators, other omega-6 and omega-3 fatty acids are released as well, such as di-homo gamma-linolenic acid (DGLA), eicosapentaenoic and docosahexaenoic acids (EPA and DHA). They are, in their turn, precursors of other lipid mediators with mainly anti-inflammatory properties, thus integrating the final inflammation and resolution responses [41]. Obviously, the result depends on the presence and balance of these fatty acids in membranes. Since recent data suggest inflammation as an important aspect in activating cancer proliferation pathways and resistance, it is evident that the membrane predisposition through its fatty acid composition is a piece of information to gather in the puzzling scenario of the cancer disease. Cancer is generated not only by genetic alterations, as a result of intrinsic or exogenous mutagens, but also by long-term exposure to acute or chronic inflammation. It is now becoming clear that the proliferation of cells alone does not cause cancer. However, sustained cell proliferation in an environment rich in inflammatory cells, growth factors and DNA-damage-promoting agents is necessary in the neoplastic process, promoting survival and migration. In this way, the causal relationship that exists between inflammation, innate immunity and cancer is more widely accepted [42]. Many of the molecular and cellular mechanisms that mediate this relationship are still unresolved, but the role that FAs play in inflammation processes related to cancer is increasingly relevant. Indeed, the role of dietary PUFAs omega-6 and omega-3 is a matter for discussion of their effects on cancer incidence and evolution [43]. The negative impact of Western diets, rich in omega-6, has recently been described in societies in which the intake of omega-6 fatty acids was traditionally in balance with that of omega-3. The number of cases with diseases associated with inflammatory processes, as well as their worse prognosis, has increased [44]. The scientific debate on the importance of the PUFA intake for cancer risk has not yet reached a conclusion. Large population studies are needed to address this task. For example, in a recent population-based (100,881 participants) prospective cohort study, using self-reported dietary data from the Västerbotten Intervention Programme, statistically significant associations have been described between a more anti-inflammatory or healthier diet and reduced risk of cancer [45]. In the development of inflammation mediated by PUFAs, both omega-6 and omega-3 FAs play crucial roles, since these two families of FAs are in constant competition with each other and, broadly speaking, they develop opposite effects. In this sense, omega-6 FAs are more related to inflammation (through the arachidonic acid (AA) cascade) and omega-3 to anti-inflammatory effects. Figure 2B shows how both omega-3 and omega-6 are closely related, by sharing the same enzymes for each step of their transformations. This fact implies that, from the beginning, there is a need for balance between the families, since, if one is favored, it will hinder the synthesis of products from the other. It is worth adding that there are regulations also from the FA-derived mediators’ formation and interactions: in the formation of prostaglandins (PG) and leukotrienes (LT) from omega-6 or omega-3 fatty acids, cyclooxygenases (COX-1, COX-2) respond more intensely for intermediates of omega-6 origin in the case of PG (PGD2, PGE2). Furthermore, not only enzymes but also receptors show different affinities, again being favored by PGs and some LTs of the omega-6 series [44]. Thus, in an environment in which omega-6 is biochemically favored, sufficient intake of omega-3 can have a key effect on the PUFA metabolism dynamics, as well as on the intensity of the action of the different eicosanoids. The involvement of PUFAs in cancer is demonstrated here by a few representative examples: regarding omega-6, AA modulates the activation of the nuclear factor kappa-light-chain-enhancer of activated B cells (NF-κB), involved in the immune response and altered in this disease. It also induces focal adhesion kinases, which promote progression and metastasis [46]. The signaling activity of AA is exerted through its transformation to PGE_2_, and, indeed, the overexpression of COX-2 protein was highlighted in several types of cancer, whereas human breast cancers frequently have high PGE_2_ levels, and breast tumors with high COX-2 protein levels are more likely to metastasize [47]. This led to consideration of COX inhibitors for cancer therapies, evaluating also their effects on angiogenesis. In this scenario, the PG interaction with EP receptors (E-series prostaglandin receptors), a family of G-protein coupled receptors designated as EP1–3 and EP4, was individuated, with the corresponding activation or deactivation of the c-AMP cascade or the extracellular signal-regulated kinase (ERK) 1 and ERK2 by way of PI3K [48]. As a matter of fact, not all the omega-6 FAs are equal in the tumor effects, and some studies suggest that, unlike the downstream omega-6 AA, the upstream omega-6s, such as linoleic acid (LA), γ-linolenic acid (GLA) and di-homo gamma-linolenic acid (DGLA), may possess anticancer effects. In fact, GLA and DGLA may exert anticancer properties via the production of PGE_1_. Although more work is needed to clarify the molecular basis of the anticancer effects of GLA and DGLA, it has been demonstrated that they are able to regulate gene and protein expression, disrupting cell-cycle progression and inducing apoptosis, a mechanism which implies also a direct effect on the lipid composition in cell membranes [48]. Regarding omega-3 PUFAs, they have the opposite effect to that mentioned for AA. For example, the combination of EPA and DHA decreases the production of eicosanoids formed from AA, leading to the inactivation of NF-κB and hindering proliferation [46]. They are also able to inhibit the activity of AKT protein, which is involved in cell survival and the inhibition of apoptotic processes [49]. Furthermore, PUFAs are involved in different processes such as lipid peroxidation, cell oxidative stress [50,51] and regulation of gene expression for controlling growth factor mediated carcinogenesis [52]. Moreover, there are other mechanisms involving lipid-based events that affect human health. The interest in ethanolamides has increased, as they are biological compounds that may have important beneficial actions by controlling inflammatory responses without being classical steroidal and non-steroidal anti-inflammatory drugs, that act by inhibiting the cascade of arachidonic acid. Palmitoyl ethanolamide (PEA) is an endogenous lipid mediator that can be found in foods (tomato, soybean, peanut) formed by palmitic acid and ethanolamide, with anti-inflammatory, neuroprotective and analgesic activities [53]. The suggested mechanisms of action involve various metabolic pathways in which some receptors seem to be activated directly (peroxisome proliferator activated receptor alpha (PPAR-α) and orphan G-protein coupled receptor 55 (GPR55)) or indirectly (cannabinoid receptors, CB_1_ and CB_2_) and transient receptor potential vanilloid type-1 channel (capsaicin receptor or TRPV1)) [53,54] to modulate mast cell activation and degranulation [53]. In colitis-associated cancer, more specifically, PEA inhibits angiogenesis through PPAR-α, suggesting a protective effect in both inflammation and cancer, being able to reduce mucosal damage, disease progression and carcinogenesis [55]. To conclude with the PUFA scenario, a brief mention of PUFA peroxidation products and the oxidative-based pathways to induce apoptosis in cancer, including ferroptosis, is made here, directing readers to reported works in these fields [56,57].

## 3. The Membrane Fatty Acid-Based Profile in Cancer and the Relevance of Erythrocytes

Considering the importance of fatty acids in membranes, a “bottom-up” approach can reveal the main changes in fatty acid profile between healthy controls and cancer patients. A systematic study of membrane fatty acid-based profiles in large populations is lacking, as is the agreement regarding the biological compartment in which fatty acids are measured. Therefore, there is large heterogeneity of data that does not currently allow us to draw conclusions about the most significant changes occurring in fatty acids in the human body. However, it is important to remark that, from the emerging scenario of fatty acids’ involvement in cancer metabolism, it is reasonable to focus the efforts towards the cell membrane compartment. In this respect, the significance of the erythrocyte cell membrane and its fatty acid composition is highlighted for several reasons: (a) the numerosity of erythrocytes and the predominance among other tissues of these cells, which constitute 70–80% of the total cells formed each day [58], rendering them the best representatives of the availability of fatty acids to construct membrane phospholipids; (b) the continuous exchange of the erythrocyte membrane phospholipids with lipoproteins and tissues in order to reshape the molecular content and satisfy homeostatic requirements [59,60]; (c) the biological mission to reach tissues and organs during the erythrocyte’s average life span of four months in humans, which requires the best performance of membrane properties in order to efficiently exchange gases; (d) the presence of the most representative SFA, MUFA and PUFA molecules and the preferred storage of arachidonic acid, known to be present in membrane phospholipids by 13–17%, as well as of other precious PUFAs [6]. Based on these considerations, the fatty acid profile can give information on the balance of these molecular components in erythrocyte phospholipids and help to establish the changes occurring under healthy and unhealthy conditions. Indeed, the fatty acid-based membrane lipidome monitoring used in different human conditions revealed how the endogenously and exogenously-derived fatty acids of erythrocytes are affected [61,62,63,64]. In Table 2, the relevant data relating to erythrocyte fatty acid monitoring from studies on cancer patients are gathered, highlighting the cancer types, the country and the number of patients and detailing the most important conclusions of each study. It is interesting to note that the SFA-MUFA transformation emerges as an important biomarker of cancer status, as well as the ratio between omega-6 and omega-3 PUFAs.

From these results, it is also clear that a large multicentric population study would definitely yield important results regarding the adoption of the fatty acid membrane profile for the follow-up of patients and therapies, assessing the importance of fatty acid biomarkers in primary and secondary prevention and discovering the molecular and clinical effects of personalized diets for cancer. We believe that the work in progress to map genetic alterations that control cell-cycle progression, apoptosis and cell growth in cancer [77] can be combined with molecular indicators such as membrane lipidomics in each tumor type to obtain more insights into the lipid pathways in cancer and clarify the epigenetic role of nutrition.

## 4. The Study of the Cancer Lipidome and the Discovery of *De Novo* Pathways: Fatty Acid Positional Isomers as New Biomarkers of Metabolic Shift

**Figure 4:**
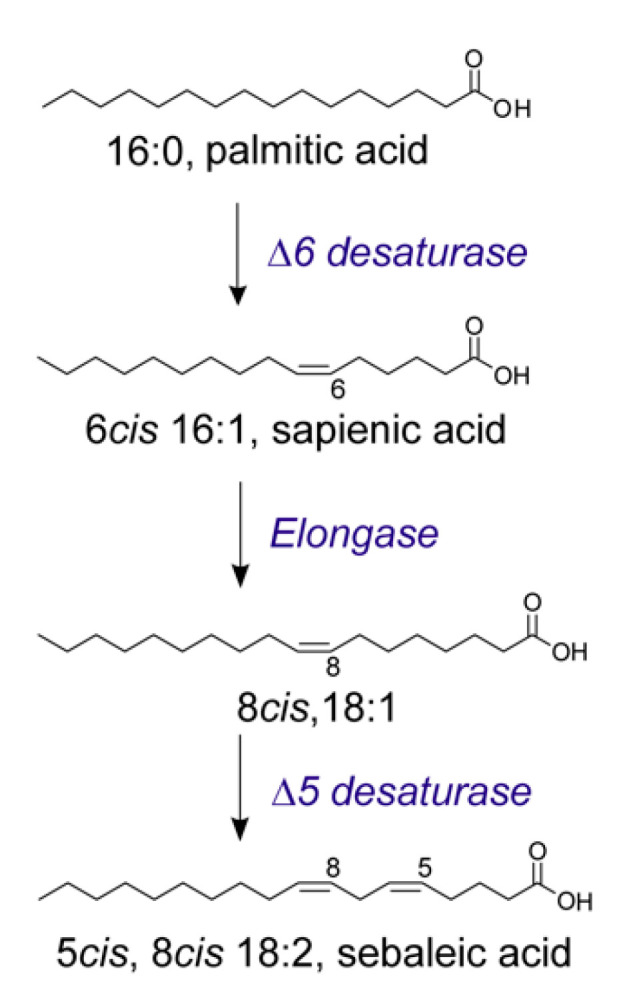
The metabolism of palmitic acid to sapienic acid (6cis-16:1) and its subsequent transformation to obtain the PUFA, sebaleic acid (5cis, 8cis-18:2).

Lipidomics in cancer helps to clarify the connections between disease and lipidome, discovering novel lipid biomarkers for diagnosis as well as alternative and synergic strategies for therapy [78]. As mentioned before, the intake of essential fatty acids (EFA) with the omega-6/omega-3 PUFA balance is of crucial importance since membranes cannot be formed without this supply. The essentiality of PUFA derives from the fact that the insertion of a second double bond in the MUFA structure cannot occur in eukaryotic cells, which means that cells do not have desaturase enzymes able to convert oleic acid (as well as palmitoleic and vaccenic acids) into PUFA in the biosynthesis (see Figure 1 and Figure 3). Since neither healthy nor cancerous cells can be formed without PUFA, it can be asked whether the dependence on dietary PUFA is a common feature and a limiting step of both types of cell metabolism. The answer to this question is not as straightforward as it seems, and in fact only recently have investigations been directed toward the study of the influence of metabolism and diet on the human lipidome. In lipidome analysis, it was also discovered that chemical skills are very important to create unambiguous protocols and distinguish fatty acid structures, especially those presenting unsaturations. A seminal example is provided by the report demonstrating for the first time of the presence of sapienic acid in various fractions of human plasma. This is a positional isomer of palmitoleic acid, which has the double bond in C6-C7 instead of C9-C10 [79]. The analytical approach for the unambiguous characterization and discrimination of positional and geometrical fatty acid isomers having the 16:1 structure is crucial for the determination of the sapienic acid presence. We described in detail the protocol of fatty acid analysis, which includes a crucial derivatization step to localize the double bond position, using the well-known dimethyl disulfide (DMDS) adducts and its diagnostic fragmentation in mass spectrometry [6,10,63,79]. It must be added that such derivatization procedure and mass spectra can be performed by regular equipment in chemical labs, and do not require specialized and expensive instrumentation. The quantitation of this fatty acid was performed in cholesteryl esters isolated from human plasma of healthy people (*n* = 5) (50.0 ± 4.0 ng/mL) and in commercially available human low density lipoprotein (LDL) samples (35.0 ± 2.0 ng/mL). How these levels are affected by health conditions in large cohorts remains to be thoroughly explored. These findings prompted us to understand in more detail the biosynthetic origin of sapienic acid. It is reported that, compared to all other types of cells that primarily form oleic acid (Figure 2A), sebocytes change their palmitic acid metabolism by the intervention of delta-6 (Δ6) desaturase enzyme (Figure 4) [80]. However, the systemic role of sapienic acid was not explored, and it was not highlighted the crucial step, that is the partition of palmitic acid between SCD-1 and delta-6 desaturase enzymes (see Figure 3). Whether this partition indicates a metabolic diversion with health significance is under current investigation. As a matter of fact, palmitic acid is an unusual substrate for delta-6 desaturase, which is an enzyme mostly involved with exogenous omega-6 and omega-3 EFA; therefore, the activation of sapienic acid biosynthesis could be attributed to several reasons, including (a) strong availability of the SFA substrate due to FAS activation, the latter well known in cancer [12,26,81]; (b) enzymatic activity competition or lack of normal intake/ presence of PUFA substrates [80,82]; (c) involvement of enzymatic polymorphism and competitive activity of desaturase for PUFA and SFA metabolisms [83]. Aiming at exploring sapienic acid and the other positional MUFA isomers in cell metabolism, we used the human colon carcinoma cell line Caco-2 to compare the results of supplementation of sapienic and palmitoleic acids (150 and 300 µM), discovering that both are rapidly incorporated into membrane phospholipids and also that the former is converted to 8cis-C18:1 and 5cis, 8cis-18:2, as depicted in Figure 4, bringing also these two fatty acids in the cell membrane phospholipid composition. The n-10 fatty acid family has a still unexplored meaning for cancer cells and we were the first to demonstrate in a cancer cell line that it involves a unique type of endogenous PUFA biosynthesis (i.e., sebaleic acid; Figure 4) leading, more importantly, to its incorporation into membranes.

The concomitant isolation of cholesteryl esters and triglycerides from the cell line demonstrated that the n-10 fatty acids “invade” all lipid classes, and, even at high concentrations (300 µM) and at long time exposures, they are not harmful (sapienic acid EC_50_ 232–265 µM for 96 h) [29]. In the same study, the biophysical properties of the cell membranes were monitored by two-photon fluorescent microscopy, using Laurdan as a dye, showing that the supplementation of sapienic acid, with respect to its positional isomer palmitoleic acid, increased fluidity in several regions, evidently correlated with the formation and distribution of n-10 MUFA and PUFA in lipid domains. Following the interest in extracellular vesicles EVs (exosomes) as relevant sites for cancer metabolism and diagnostics [84], we investigated the presence of the n-10 fatty acid family, comparing membrane phospholipids and EVs of prostate cancer cell lines with different degrees of aggressiveness: PC3 (prostate cancer) and LNCaP (prostate derived from metastatic site: left supraclavicular lymph node), the former being more aggressive [10]. We found that 12–13% of the membrane fatty acids of these cell lines were composed of n-10 fatty acids, with the sapienic acid content >7%. In EVs, n-10 fatty acids were 9% for PC3 EVs and 13% for LNCaP EVs, with statistically significant increases in 8cis-18:1 and 5cis, 8cis-18:2, which is relevant considering that the EV are involved also in the transport of biologically active lipids and lipid metabolites to feed cancer tissues. This discovery can have a strong impact also in cancer diagnostics and follow-up of intervention efficacy. We envisaged that the sapienic/pamitoleic ratio, found equal to 3.5 in prostate cancer cells, also provides a measure of the partition into two metabolic pathways, and, in these cell lines, the delta-6 desaturase transformation of palmitic acid was found to be unusually high. A parallel evaluation of gene expression for desaturase (FADS) and elongase (ELOVL) enzymes by qRT-PCR (quantitative real time polymerase chain reaction) evidenced significant increases in FADS expression in PC3 with respect to LNCaP cells, and the higher expression of ELOVL5 in PC3 compared to LNCaP cells with ELOVL6 significantly lower. We found interesting evidence of higher desaturase activity in the most aggressive PC3 cell line, and suggested deepening the study of FADS3 desaturase, which, so far, has an uncertain metabolic role [85]. Indeed, the role of desaturase enzymes represents an important aspect in cancer metabolism and is also considered as a target in anticancer therapy, as reported in reviews [86] showing these strategies applied in preclinical trials. Regarding elongases, most of them are tumor specific: for example, ELOVL1, ELOVL5, ELOVL6 and ELOVL2 are highly expressed in breast cancer [87,88] and ELOVL7 in prostate cancer [89]. In the new scenario of the n-10 fatty acid family, a recent work confirmed the presence of sapienic acid in different cancer cell lines, defining it as a contributor to cancer plasticity [30], and another paper reported an increase in the transformation of palmitic acid to sapienic acid induced by the increase in mammalian target of rapamycin (mTOR) and sterol regulatory element-binding protein 1 (SREBP-1) signaling in mouse embryonic fibroblasts (MEFs) and U87 glioblastoma cells [90]. In this report, the inhibition of the two signaling pathways led to a decrease in sapienic acid biosynthesis. On the other hand, it must be recalled that fatty acids’ enzymatic activities can be influenced by dietary fats, as previously shown for the competition between palmitic acid and PUFA omega-6 and omega-3 precursors [91].

New pathways involving SFA and MUFA are going to be discovered, provided that analytical protocols are able to give satisfactory results, such as was recently shown by the transformation of oleic acid in MCF7 cell lines into an eicosanoic fatty acid (7cis, 11cis-20:2) obtained by the unusual activity of FADS1 desaturase introducing a double bond at the level of C7 and not C5 [92]. Considering all the published work on the subject, so far only our experiments with cancer cell lines demonstrated the new pathway that brings about endogenous PUFA synthesis (sebaleic acid) and determined the n-10 FA insertion at the level of membrane phospholipids. We believe that this outcome of the sapienate metabolism is the real contribution to cancer plasticity, strongly influencing fluidity changes that are deeply embedded in cancer signaling and metabolism. We envisage that the pathway of sapienic acid will have a strong development in metabolic, therapeutic and nutritional research; here, we have provided a careful literature summary of the various contributions available so far, that we hope will be useful to researchers interested in the field.

## 5. Link between Obesity and Cancer: When the Lipid Supply Becomes Dangerous

Despite the difficulty of definitively proving that obesity is one of the causes of cancer, it remains a recognized risk factor contributing to the development and progression of tumors [93]. Several observational studies evidenced that obese and overweight subjects have a higher risk of developing cancer than lean subjects; in 2016, the International Agency for Research on Cancer (IARC) declared that obesity was associated with an increased risk for 13 types of cancer, indicated in Table 3 with their corresponding epidemiological studies [94,95].

**Figure 5:**
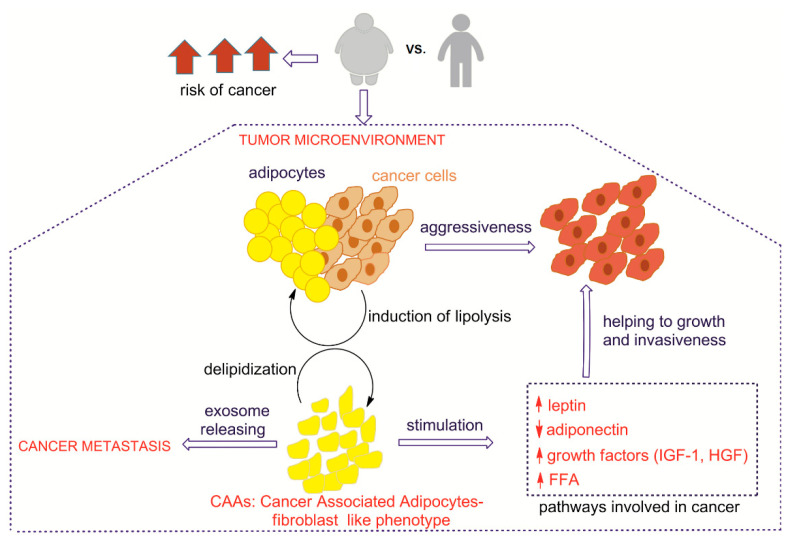
Relevant metabolic connections between adipose and cancer tissues; the arrow ↑ means increase, the arrow↓ means decrease.

The clarification of the mechanisms binding obesity to cancer is crucial for the diagnosis and implementation of effective therapies. Here, we have gathered the main molecular pathways connecting adipose tissue (AT) and adipocytes with cancer cells in the tumor microenvironment, as well as their impact on cancer growth, invasion and metastasis. We summarize relevant connections between adipose and cancer tissues in Figure 5.

The close localization between adipose tissue and cancer cells, immoderately increased in obese subjects due to the effect of excess of calories not consumed, induces a deep modification of the phenotype and functioning of adipocytes, which become cancer-associated adipocytes (CAA), promoted for the induction of lipolysis by cancer cells. Adipocytes are decreased in number and size, showing delipidization and de-differentiation to fibroblast-like phenotype [128,129,130,131]. It is well known that the exposure of adipocytes to cancer cells for long periods, with consequent fibroblast morphology, induces the formation of cancer cell fibroblast populations that are involved in tumor invasiveness [132]. In this context, the transformation of adipocytes provides a total alteration of their secretory function involved in endocrine, metabolic and immune systems. The ways in which the specific fatty acid status of adipocytes is involved in the support of cancer cells growth and metastasis are not yet well defined. The identification of several fatty acid unbalances in the erythrocyte membranes of obese patients certainly highlights derangements of lipid metabolism, including the above mentioned sapienic acid pathway [63]. Increased release of free fatty acids accompanies altered levels of adipokines and pro-inflammatory cytokines, growth factors and hormones [133]. The signaling in several types of cancer cells is sustained by adipokines secreted by adipocytes, mainly including leptin, adiponectin, oestrogens, insulin-like growth factor 1 (IGF-1) and hepatocyte growth factor (HGF). In particular, the leptin/adiponectin ratio can be an interesting value to examine, due to the opposite effects of these hormones. Leptin stimulates a cascade of signaling events inducing JAK2/STATs, MAPK/ERK 1/2, PI3K/AKT and PKC, JNK, p38 MAPK and AMPK pathways in diverse cellular types (see abbreviations) [134]. The mechanism implicates the interaction with transmembrane leptin receptor (LRb) that, if phosphorylated, mediates downstream LRb signaling controlling STAT3 (signal transducer and activator of transcription 3) and ERK activation [135,136]. Simultaneously, high levels of leptin induce the stimulation of monocytes into macrophages, leading to chronic, obesity-associated inflammation. Leptin increases the expression of anti-apoptotic proteins, inflammatory markers (tumor necrosis factor, TNF-α, interleukin IL-6) and angiogenic factors (vascular endothelial growth factor, VEGF), all processes involved in cancer cell survival, proliferation and migration [137,138]. On the other hand, adiponectin is inversely correlated to the body mass and cancer, inducing apoptosis and decreasing tumor vascularization. It modulates multiple signaling pathways, exerting its physiological and protective functions through the receptors AdipoR1 and AdipoR2 [139,140,141]. It is also able to block angiogenesis, inhibiting endothelial cell proliferation induced by FGF2 (fibroblast growth factor 2) as well as the migration of endothelial cells by VEGF. Furthermore, adiponectin inhibits cancer growth and proliferation, interfering with several pathways like AMPK, MAPK and PI3K/AKT, ERK1/2-MAPK pathway and GSK3/catenin, inducing G0/G1 cell-cycle arrest [142,143,144,145]. Adiponectin-induced cell death is also accompanied by an increase in intracellular reactive oxygen species (ROS). As a matter of fact, adiponectin pre-treatment suppresses leptin-induced ERK and AKT signaling [146]. Here, we only mention the roles of insulin and glucose levels and their interactions with specific receptors, such as insulin-like growth factor 1 (IGF-1) and hepatocyte growth factor (HGF), that correlate with increased risks of specific cancers, like ovarian and breast cancers, mainly through activation of PI3K/AKT and MAPK pathways [147], while the inhibition of IGF-IR kinase activity prevents the growth-promoting effect of adipocytes on breast cancer cells [148]. Additional factors are connected with the altered environment of adipose tissue and cancer for the increase in inflammatory conditions, with consequent liberation of pro-inflammatory mediators, among them TNF-α and IL-6, contributing to the growth and differentiation in tumors like lymphoma, pancreatic and liver cancers. TNF-α induces carcinogenesis, activating the nuclear transcription factor NF-κB that prevents apoptosis, allowing enhanced cell survival, growth and proliferation [134,149]. IL-6 is normally elevated in obesity, induces JAK-STAT3 signal transduction and, stimulates cell proliferation, differentiation and metastasis. It mediates cell proliferation through the MAPK pathway; in fact, in some studies, the inhibition of MAPK stopped proliferation in the presence of IL-6, evidencing the role of cytokines in cell proliferation connected with inflammation [150]. Inflammation signaling, as discussed in Section 3, is an important piece of information to acquire in order to estimate factors that trigger cancer and its progression. The need for an integrated metabolic scenario emerges, linking the balance of membrane fatty acid precursors of eicosanoids and other lipid mediators with the effects of fat accumulation and hormonal control.

The effect of transformation of adipocytes in CAAs is evidenced also by increased release of free fatty acids (FFA), with the immediate effect of generating energy to fuel tumor growth [151]. The mobilization of FFA from adipocytes is performed in three steps by lipase enzymes: ATGL (adipose triglycerides lipase), HSL (hormone sensitive lipase) and MAGL (mono acylglycerol lipase), enhancing their circulating levels [93]. Seminal experiments of co-culture of adipocytes with cancer cells showed that there is stimulation of lipolysis in adipocytes releasing FFA and glycerol, with a reduction in adipocyte size [152]. The amount of FFA promoted cancer progression, delivering building blocks for cancer cells but also stimulating lipid metabolism; in ovarian cancer cells, co-cultures with adipocytes induced upregulation of fatty acid β-oxidation (FAO), with a consequent large quantity of ATP [133,139,153,154], supporting the energy demand of the tumor mass. The transfer process of FFA between adipocytes and cancer cells is mediated by fatty acid-binding protein 4 (FABP4), which supplies energy to the cells and also active oncogenic pathways like IL-6/STAT3/ALDH1, leading to an enhanced stem cell-like phenotype and tumor progression [133]; FABP4 expression increased in cancer cells co-cultivated with adipocytes [155]. The interesting connections of disease development with the fatty acid structures and functions discussed in the previous sections should appear clear at this point, and this review has the scope of stimulating a constructive debate among scientists involved in cancer cell biology, metabolomics and lipidomics in order to use the substantial information available to develop lipid-based diagnostics and strategies for cancer.

The recent results obtained with EVs in cancer offer promising perspectives on mechanistic and diagnostic developments [156]. The transport of lipids by EV from AT can be an important player in the whole scenario. In a case study of melanoma, the biomolecular transfer process of EVs seems to increase in the presence of obesity. Incubation of melanoma cells with EVs deriving from AT caused the redistribution of lipid droplets close to mitochondria and the increase of fatty acid oxidation [157]. Research conducted on overweight subjects showed that exosomes derived from cancer cells were incorporated by adipocytes, modifying transcriptome and cytokine secretion; the exosomes obtained from adipocytes strongly helped tumor growth by angiogenesis and enhanced inflammation, recruiting macrophages, activating kinases and involving the NF-κB signaling pathway [158,159]. Together with the analysis of the fatty acid types contained in EVs, including sapienic and sebaleic acids, the EVs have enormous potential for unveiling new aspects of lipid supply to cancer.

Obesity also influences the effects of anticancer therapies, as shown in obese cancer patients compared with non-obese patients evaluated for the effects of the same drug treatment [160]. Besides the various aspects involved in these effects, it is important to highlight that the fatty acid constituents of adipose tissue assume a fundamental role in the modification of pharmacokinetics, conferring drug resistance [161,162].

In the scenario of lipid metabolism, the role of lipophagy (i.e., autophagic degradation of lipid droplets, the main lipid storage organelles of eukaryotic cells), discovered in 2009 to have important consequences on health [163], must be mentioned here, in connection with the ongoing debate concerning the role of fasting strategies in cancer treatment [164]. The ways in which calorie restriction/control impacts obesity and cancer treatment will be matter for further research and active debates from different perspectives [165].

## 6. Some Considerations of Fatty Acid-Based Membrane Lipidomics and Lipid Therapy

Tumoral cells develop accelerated *de novo* lipogenesis as well as strong lipid recruitment, also taking advantage of obesity, to sustain their needs. However, the quality of fatty acids contributes to their invasiveness, also due to their influence on the biophysical properties of membranes and signaling cascades. The proposal of the “membrane hypothesis” links the initial steps of death or life of tumoral cells with the moment of the phospholipid aggregation for membrane formation and the balance between the saturated and unsaturated fatty acid types present in the individual. This crucial balance is different from tissue to tissue, since each tissue has its own composition [7], and it is important to remark that the membrane formation is a completely spontaneous process of phospholipid aggregation, which in their turn are formed by the availability of fatty acids in the lipid pools. It could be said that, with respect to the adequate intake (AI) of lipids established by the main international agencies of health and food [166], the lipid pool should be able to reach a satisfactory balance, with scarce possibilities for impairment or excess. We are aware of the strong ongoing debate about the interplay between genetics and other causes of cancer [167,168,169,170]; however, we wish to highlight the importance of the environment (including nutrition), able to interfere with fatty acid levels and metabolic transformations, with strong impact on inflammatory responses and stress conditions, including on hormonal effects such as explained in obesity (Section 5), that can change the “normal” scenario and create unbalances. In our opinion, it is timely to introduce the monitoring of SFA, MUFA and PUFA membrane levels in clinical practice, in view of evaluating strategies that influence the formation of membranes in the individual. Fatty acid-based membrane lipidomics can give the necessary information to estimate the correctness of the molecular pool, which is the *conditio sine qua non* for the healthy behavior of this important compartment [61]. It is worth recalling that the erythrocyte membrane compositions of patients under parenteral nutrition reflected the lipid emulsions given to them. In particular, olive oil emulsion was able to induce statistically significantly higher levels of arachidonic acid and omega-6/omega-3 ratio compared to patients treated with a lipid emulsion containing a small percentage of fish oil [64]. This is an important message for those involved in patient care and nutrition and also for considering the exact dosage of fatty acid supplementations for therapeutic purposes. As a matter of fact, membrane homeostasis and related therapies are nowadays emerging, targeting cell membranes by dietary bioactive molecules able to obtain the remodeling of plasma membrane domains. The attenuation of oncogenic protein activity by modulating the membrane organization of essential proteins and lipids was proven and this is a promising way to use such an approach to manage cancer expansion. It is worth underlining that omega-3 has a very potent influence on membrane organization and this ability, combined with the anti-inflammatory activity, should be developed toward successful cancer treatments [171,172]. However, it is also evident that, without assessing the membrane status in the individual, the assignment of the lipid strategy cannot be precise, in types and doses, thus even bringing about contrasting clinical outcomes since the membrane unbalance results or remains altered. Therefore, it will be necessary to develop a multidisciplinary approach, involving also clinicians, for the understanding of membrane molecular profiles and for creating protocols of membrane lipidomics and lipid therapy, gathering evidence-based results. In this direction, lipid replacement therapy (LRT) is described as a natural medicine approach to replace damaged lipids in cellular membranes and organelles; however, no personalization is proposed [173]. In the context of membrane therapy, we must also mention natural fatty acids with a structure able to interfere with lipid enzymes, such as sterculic acid, a cyclopropane-containing derivative of oleic acid (9,10-methylene-9-octadecenoic acid) found in plants of the genus *Sterculia*. This is an inhibitor of SCD-1, and of the related cascades, as previously explained, which has attracted interest for application in various diseases, including cancer [174]. As previously described, lipid enzyme inhibitors (fatty acid synthesis and desaturation) are attracting interest for innovative cancer treatments, and readers are directed to reviews to deepen the state-of-the-art of such therapeutic strategies [15,21,174].

Focusing on mature erythrocytes, their membrane composition data can be gathered from cancer patients, accompanying the biological sample with an accurate food questionnaire. By this approach, we were able to highlight in a preliminary study of cancer patients that they have SFA-MUFA membrane levels which are significantly different from controls and independent of dietary intakes [69]. It is also straightforward that the analytical protocols used for membrane lipidomic analysis must be certified by international accreditation bodies, and it is advisable that such protocols are unified and automatized by high-throughput procedures, in order that clinical laboratories can gather reliable “big data” to depict cancer lipidomics in an incontrovertible manner.

## 7. Conclusions

The acquisition of a multidisciplinary vision of fatty acids’ relevance to membrane formation and cancer development is necessary in order to go from the bench to the bedside and to the home of patients, associating nutrient choice with strategies to defeat cancer. The growing understanding of the response of cancer to diet will lead to new therapeutic opportunities but, at the same time, will have practical use in the everyday lives of patients, solving also contrasting effects reported in the literature for PUFA supplementation [175]. It is desirable to increase efforts for a larger understanding of molecular nutrition effects in combination with pharmacology and immunology to control this multifaceted disease [176]. Researchers of several disciplines are required in order to accomplish such goals. Specific effort is needed by clinical units to introduce fatty acid diagnostics tools and therapies to prove the validity of the concepts and translate them into medical practice. Indeed, previously reported clinical effects for some fatty acids, such as the omega-6 γ-linolenic acid (see Figure 2), of its antitumoral synergy with chemotherapy [177] must take into account its rare presence in foods and be evaluated in a personalized way, also determining the level of this fatty acid in the individual. Therefore, knowledge of molecular diagnostics, such as membrane lipidomics, is a fundamental step toward including endogenous and exogenous fatty acids in the cancer scenario.

## Figures and Tables

**Table 1:** The main fatty acid-driven mechanisms and biological effects in cancer onset, growth and metastasis.

Entry	Implicated Mechanism	Biological Effects	Lit
1	Desaturation from 16:0 to 6c-16:1 (sapienic acid)	Support of membrane biosynthesis during proliferation	[29,30]
2	mTORC2 regulation of lipid metabolism	Glycolysis and lipogenesis activation	[31,32]
3	Acetyl CoA synthetase 2 promotion of acetate utilization	Maintaining cancer cell growth under hypoxia and metabolic stress	[33]
4	Adipokines mediation of ovarian cancer metastasis	Induction of lipolysis and β-oxidation to provide energy	[34]
5	Enhanced uptake of exogenous lipoproteins	(a) Cholesteryl ester accumulation, induced by PTEN loss and PI3K/AKT activation, to sustain cancer aggressiveness (b) Increased amount of cholesterol and overexpression of low-density lipoprotein receptor to boost proliferation (c) Sustaining proliferation and aggressive potential of breast cancer tumors	[35] [36] [37]
6	Increase in lipid droplets in tumor cells	Increased COX-2 expression and storage in droplets, with effects on proliferation	[38]
7	Stearoyl CoA desaturase essentiality for cancer cell survival	Inhibition of FA desaturation, blocking the synthesis of lipids and impairing cell survival	[39]

**Table 2:** Fatty acids in cancer: collection of data from studies on erythrocyte membrane fatty acids in patients affected by different types of cancer and emerging biomarkers.

Cancer Type	Country	Human Cohort Size	Outcomes	Reference
Breast/Prostate/Liver/Pancreas/Colon/Lung	Puerto Rico	255 cancer patients 2800 non cancer patients 34 healthy volunteers	Lower levels of stearic acid and increased content of oleic acid. EPA and DHA/ALA ratio to estimate PUFA imbalances in cancer patients.	[65]
Colorectal	Japan	61 cases 42 controls	Less EPA and linoleic acid and high levels of arachidonic acid in cancer patients.	[66]
Breast	Italy	71 cases 141 controls	High oleic acid and low stearic acid in patients. Oleic acid and MUFA positively associated with breast cancer risk. Saturation index (stearic/oleic acids ratio) inversely correlated.	[67]
Colorectal	Italy	13 cancer patients 13 patients with no malignant diseases	Lower levels of n-3 PUFAs and higher n-6/n-3 PUFA ratio in cancer patients.	[68]
Breast/Colon/Lung	Spain	54 cases 34 controls	Less SFA (C16:0 and C18:0), high MUFA (9c-C18:1 and 11c-C18:1) compared to controls. In the PUFA families, increase in n-6 C18:2 and C20:3 (15.7% and 22.2%, respectively).	[69]
Colorectal	France	328 cases 619 controls	High levels of pentadecanoic and heptadecanoic acids; oleic acid and linoleic acid associated with the risk of advanced adenomas. EPA and DHA negatively associated with the risk of advanced adenomas.	[70]
Basal Cell Carcinoma	Iran	40 cases, 40 controls	Low palmitic and high oleic acid levels in cancer patients. Saturation index (stearic/oleic acids ratio) lower in cancer patients.	[71]
Basal Cell Carcinoma	Iran	40 cases, 40 controls	Higher AA, total omega-6 and LA in cancer patients, lower omega-3.	[72]
Colorectal	Japan	74 cases, 221 controls	Risk of colorectal cancer inversely associated with DHA, AA and PUFAs and positively associated with palmitic acid, SFAs and SFA/PUFA.	[73]
Breast	China	322 cases, 1030 controls	Significant direct association among palmitic, γ-linolenic, palmitoleic and vaccenic acids and risk of breast cancer. Total n-3 fatty acids, EPA and 16:0/16:1 saturation index associated with significantly lower risk of breast cancer.	[74]
Prostate	USA	127 cases, 183 controls	MUFA and α-linolenic/EPA ratio associated with reduced risk of prostate cancer.	[75]
Advanced squamous cell lung carcinoma (SCC), lung adenocarcinoma (ADC) and small cell lung cancer (SCLC)	Spain	63 patients, 50 controls	AA, EPA, palmitic, oleic acids biomarkers in diagnosis and in other aspects related to clinical disease management of cancer.	[76]

**Table 3:** Increased risk for 13 cancer types correlated to overweight/obesity (% increased risk OW/OB vs. lean) and their corresponding epidemiological studies.

Cancer Type	Increased Risk (OW/OB vs. Lean)	References
Endometrial	150–200%	[96,97]
Esophageal	200–400%	[98,99]
Gastric cardia	168–188%	[100,101]
Liver	17–89%	[102,103,104]
Kidney	200%	[105,106,107]
Multiple myeloma	10–20%	[108,109,110]
Meningioma	10–20%	[111,112]
Pancreatic	50–60%	[113,114]
Colorectal	30–60%	[115,116,117]
Gallbladder	20–60%	[118,119]
Breast	20–40%	[120,121,122,123]
Ovarian	10–30%	[97,124]
Thyroid	10–30%	[125,126,127]

## Abbreviations

AKT	Protein kinase B
AMPK	5′ adenosine monophosphate-activated protein kinase
AT	Adipose tissue
ATGL	Adipose triglycerides lipase
ATP	Adenosine triphosphate
CAAs	Cancer-associated adipocytes
DMDS	Dimethyl disulfide
EFA	Essential fatty acids
ERK	Extracellular signal-regulated kinases
EVs	Extracellular vesicles
FABP	Fatty acid binding protein
FADS	Fatty acid desaturase
FAO	Fatty acid oxidase
FFA	Free fatty acids
FGF2	Fibroblast growth factor 2
GSK3	Glycogen synthase kinase 3 beta
HGF	Hepatocyte growth factor
HSL	Hormone sensitive lipase
IGF-1	Insulin growth factor 1
IL-6	Interleukin 6
JAK2	Janus kinases 2
JNK	c-Jun N-terminal kinases
LDL	Low density lipoproteins
LNCAP	Prostate derived from metastatic site
LR	Leptin receptor
MAGL	Mono acylglycerol lipase
MAPK	Mitogen-activated protein kinases
MEFs	Mouse embryonic fibroblasts
PC3	Prostate cancer
PI3K	Phosphoinositide 3-kinases
PKC	Protein kinase C
PPAR	Peroxisome proliferator activated receptor
SREBP-1	Sterol regulatory element- binding protein 1
STAT	Signal transducer and activator of transcription protein
STAT3	Signal transducer and activator of transcription 3
TNF-α	Tumor necrosis factor alpha
VEGF	Vascular endothelial growth factor
